# Characterization of Plaque-Sized Variants of Daniel’s (DA) Strain in Theiler’s Virus-Induced Epilepsy

**DOI:** 10.1038/s41598-019-38967-z

**Published:** 2019-03-05

**Authors:** M. Bijalwan, C. R. Young, J. Tingling, X. J. Zhou, A. R. Rimmelin, J. L. Leibowitz, C. J. Welsh

**Affiliations:** 1 Veterinary Integrative Biosciences, College of Veterinary Medicine and Biomedical Sciences, College Station, Texas USA; 2 Microbial Pathogenesis and Immunology, Texas A&M Health Science Center, College Station, Texas USA; 3 Texas A&M Institute for Neuroscience, Texas A&M University, College Station, Texas USA; 4 College Station High School, Texas A&M Health Science Center, College Station, Texas USA; 5 Women’s Health in Neuroscience Program, Texas A&M Health Science Center, College Station, Texas USA

## Abstract

Epilepsy is a complex neurological disease characterized by recurrent seizures. Patients with viral encephalitis have a 16-fold increased risk of developing epilepsy, and this risk can persist for about 15 years after the occurrence of initial viral infection. Theiler’s murine encephalomyelitis virus (TMEV) infection induces a well-characterized experimental model of epilepsy in C57BL/6 mice. In response to intracerebral (I.C.) injection of Daniel’s (DA) strain of TMEV, there is vigorous immune response, which is detrimental to neurons and contributes to acute seizures, rendering mice susceptible to epilepsy. A comparative *in vivo* challenge study with either one of the two variants of the DA strain, small (DA-D_S_) or large (DA-C_L_) plaque forming variants, revealed differences in the diseases they induced in C57BL/6 mice. Compared to DA-C_L_-, DA-D_S_-infected mice exhibited significantly more seizures, higher clinical scores, neuroinflammation, and neuronal damage (mainly in the CA1-CA2 regions of hippocampus). Moreover, the brains of DA-D_S_ infected mice contained approximately five-fold higher virus than those of DA-C_L_ infected mice. A sequence comparison of the DA-C_L_ and DA-D_S_ genome sequences showed mutations in the leader (L) and L* proteins of DA-C_L_ variant, which may be the cause of attenuating phenotype of DA-C_L_ variant in the C57BL/6 mouse model of epilepsy.

## Introduction

Theiler’s murine encephalomyelitis virus (TMEV) is a single stranded RNA virus that belongs to the *Picornaviridae* family^1^. It is found naturally in the enteric system of mice^2,3^. TMEV is divided into two serologically related but biologically and neuropathologically distinct subgroups, GDVII and Theiler’s original (TO). The GDVII subgroup contains highly neurovirulent GDVII and FA strains, while the TO subgroup contains less neurovirulent DA, BeAn 8386 (BeAn), WW, Yale, and TO4 strains^1,2,4–7^.

Intracerebral (I.C.) infection with TMEV induces different neurological diseases in mice based upon the virus strain or mouse strain used^1,4,8–15^. The GDVII subgroup causes acute fatal polioencephalomyelitis in all strains of mice including SJL mice^4,8^, and acute fatal encephalitis accompanied with seizures in C57BL/6 mice^13,14^. DA and BeAn are the two most commonly studied virus strains in the TO subgroup. DA- or BeAn- infected SJL mice develop biphasic disease characterized by early (weeks 1–2 post-infection [p.i.]) mild poliomyelitis and late (~2 months p.i.) demyelinating disease^1,10^. In SJL mice, infectious virus is not cleared and persists at low titers, primarily in the spinal cord^1,16–18^. In contrast to developing demyelinating disease, DA- or BeAn- infected C57BL/6 mice develop acute (within a week p.i.) seizures^13^ that progress into epilepsy after an indefinite latent phase (~1–2 months p.i.)^19^. In C57BL/6 mice infectious virus is cleared from the CNS within one month p.i.^20^. Interestingly, tonic-clonic seizures or hyperexcitabilty were also observed in FA-, and occasionally in GDVII- infected Swiss mice^8^. However, like the SJL mice, DA-infected Swiss mice develop demyelinating disease^9,11^. This suggests some phenotypic overlap among the TMEV strains, and among the mouse strains.

TMEV subgroups also differ from one another based upon their *in vitro* characteristics. The GDVII viruses form large plaques (1 to 5 mm), whereas the TO viruses usually form small plaques (0.2–1 mm) on baby hamster kidney (BHK) cells^4,5^. Dr. Leibowitz and colleagues observed that their DA strain of TMEV produced a mixture of large and small sized plaques when assayed on L2 cells. The large plaques were 1.51 ± 0.16 mm, while small plaques were 0.75 ± 0.13 mm in size. The genetically stable, large and small sized plaques forming variants of the DA strain were isolated and called DA-C_L_ and DA-D_S_, respectively^21^.

The growth kinetics and neurovirulence of DA-C_L_ and DA-D_S_ plaque variants have been well documented in TMEV-induced demyelinating disease. The DA-C_L_ variant yields higher titers *in vitro*, but replicates to lower titers in the CNS and is unable to induce any clinical disease in SJL mice even though it is able to persist in the CNS. In contrast, the DA-D_S_ variant yields lower titers *in vitro*, but replicates efficiently in the CNS and induces demyelinating disease in SJL mice. Following infection with DA-D_S_, there is higher viral growth in the CNS and increased severity of demyelinating disease compared to the parental DA virus^21^. However, no further studies have been carried out to determine the relative virulence of these DA variants in the TMEV-induced epilepsy model.

The TMEV-induced epilepsy model is the first viral infection-driven animal model developed that provides us with the opportunity to understand both the acute and chronic forms of seizures. Infection in C57BL/6 mice with the DA or BeAn strain of TMEV stimulates a vigorous host immune response (central and peripheral) that targets the virus but could also cause damage to the pyramidal neurons of the hippocampus in the process of clearing infection. The unregulated host immune response and neuronal damage triggers hippocampal excitability, acute seizures, and reduced seizure threshold. The acute phase events pave the way for the development of recurrent spontaneous seizures (epilepsy) in the chronic phase^13,19,22–25^.

Here, we identified the genetic differences between the two plaque variants, DA-C_L_ and DA-D_S_, and characterized their neurovirulence and pathogenesis in the epilepsy model. The DA-C_L_ variant was relatively avirulent as it induced reduced neuroinflammation, neuropathology and minimal seizures in infected mice. Conversely, the DA-D_S_ variant was highly neurovirulent as it provoked pronounced neuroinflammation, hippocampal damage and seizures in infected mice. Thus, DA-C_L_ was attenuated in both the epilepsy and late demyelinating models. Moreover, our results suggest that the relative neurovirulence of the DA variants is independent of the genotype of SJL and C57BL/6 mouse strain. A sequence comparison of the DA-C_L_ and DA-D_S_ genome sequences revealed two differences between the two variants resulting in three coding differences at the protein level, in the Leader (L), L*, and 2C proteins.

## Results

### Seizures induced by infection with DA-D_S_ were significantly more frequent and more severe than those induced by DA-C_L_

**Figure 1:**
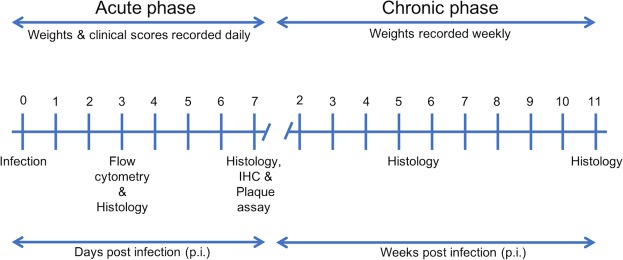
Experimental timeline. The acute phase of disease is defined as up to day 7 p.i., during which seizures, non-epileptic clinical illness, weights, viral burden, inflammation and CNS pathology were determined. The chronic phase is defined as from week 2 to 11 p.i. or until the termination of mice. Chronic phase measures included weights, and histological analyses of the CNS.

**Figure 2:**
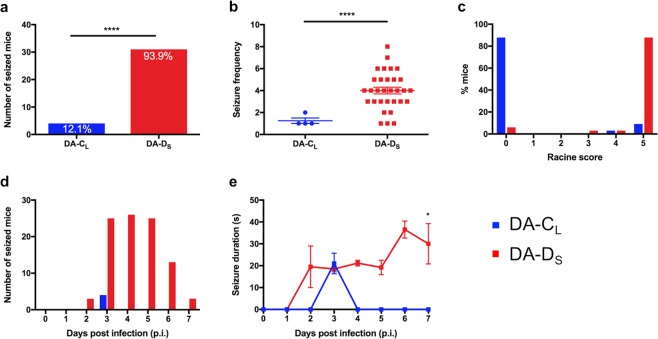
Seizures induced by infection with DA-D_S_ were significantly more frequent and more severe than those induced by DA-C_L_. To record seizures among infected groups, mice were observed twice a day for a week following infection. (**a**) The % of seized mice in each group was calculated as (number of seized mice/total number of infected mice) × 100 (****p < 0.0001 by Fisher’s exact test). (**b**) The seizure frequency was calculated as number of seizures recorded per mouse during acute phase (****p < 0.0001 by t test with Welch’s correction). The data are only from seized mice in DA-C_L_ and DA-D_S_ groups, N = 4 and 31, respectively. (**c**) The severity of seizures was determined based on the Racine scoring system, where score 0 indicated no seizure occurrence, and score 5 indicated tonic-clonic seizures. (**d**) The number of seized mice (**e**) and the duration of seizures were recorded each day until day 7 p.i. (*p < 0.05 by Repeated measures one-way ANOVA with post test for linear trend). Graphs (**a**–**d**) show pooled results from four separate experiments expressed as number/percent/mean ± SEM, N = 33 per infected group. Graph (**e**) shows pooled results from three separate experiments expressed as mean ± SEM, the data are from seized mice in DA-C_L_ and DA-D_S_ groups, N = 3 and 22, respectively.

Groups of 4-week-old C57BL/6 mice were infected with DA-C_L_, DA-D_S_, or mock-infected by I.C. injection as described in Materials and Methods (Fig. 1). To record seizures, we observed mice twice a day (mornings and evenings) for a week following infection. During the first 7 days of infection, mice infected with the DA-D_S_ variant had a significantly higher incidence of seizures (31/33 mice) than the DA-C_L_ variant (4/33) (Fig. 2a) (Fisher’s exact test, p < 0.0001). No seizures were found in the control group (not shown).

The infected mice usually seized within the first few minutes of observation, suggesting that handling-associated stress, moving the cages from the racks to the hood, removing the cage lids when the cages were inside the laminar flow hood, placing mice in the hood for monitoring seizures/flaccid paralysis, etc. played an important role in triggering seizures. Seizures occurred both in the mornings and in evenings suggesting no association between seizures and circadian rhythm. Moreover, 64% of the DA-D_S_-infected mice experienced more than one seizure during the day, compared to only 3% in the DA-C_L_- infected group. Additionally, seized mice were individually monitored for the total number of seizures (frequency) during the acute phase (Fig. 2b). The total number of seizures observed was significantly higher in the DA-D_S_-infected group (Mean ± SEM = 4 ± 0.31) compared to that in the DA-C_L_-infected group (Mean ± SEM = 1.25 ± 0.25) (t test with Welch’s correction; t(15.42) = 6.94, p < 0.0001).

The intensity of seizures was greater in DA-D_S_-infected mice with approximately 88% of the mice exhibiting Racine stage 5 level seizure activities, whereas only 9% of the DA-C_L_-infected mice had seizures of that severity (Fig. 2c). These severe seizures started with head nodding and rapidly progressed to tonic-clonic seizures. Seizures in the DA-D_S_-infected group started from day 2 p.i., the majority of seizures occurred on days 3, 4 and 5 p.i., and seizures completely ceased after day 7 p.i. (Fig. 2d). In comparison, seizures in the DA-C_L_-infected group occurred only on day 3 p.i. The duration of seizures in the DA-D_S_-infected group significantly increased over time (Repeated measures one way ANOVA; F(5, 57) = 4.96, p < 0.001), where the Mean ± SEM escalated from 19.5 ± 9.5 on day 2 p.i. to 30 ± 9.24 on day 7 p.i. Moreover, the duration of seizures showed a significant linear trend (Post test for linear trend; F(1, 57) = 5.376, p =  < 0.05) (Fig. 2e). However, the duration of seizures that occurred on day 3 p.i. was not significantly different between DA-D_S_-infected group (Mean ± SEM = 18.47 ± 1.32) and DA-C_L_-infected group (Mean ± SEM = 21.00 ± 4.72) (Fig. 2e) (t test with Welch’s correction; t(2.32) = 0.52, p > 0.05).

To confirm our findings, we video-recorded nine mice from each infected group 24/7 for a week, and the rater analyzed the videos without prior knowledge of the experimental conditions. As expected, we did not find any seizures in the DA-C_L_-infected groups, while a total of 8 seizure episodes, Racine stage 4 or 5, were recorded in the DA-D_S_-infected groups during the acute phase.

### Mice infected with DA-D_S_ plaque variant displayed severe non-epileptic clinical signs in the acute phase

**Figure 3:**
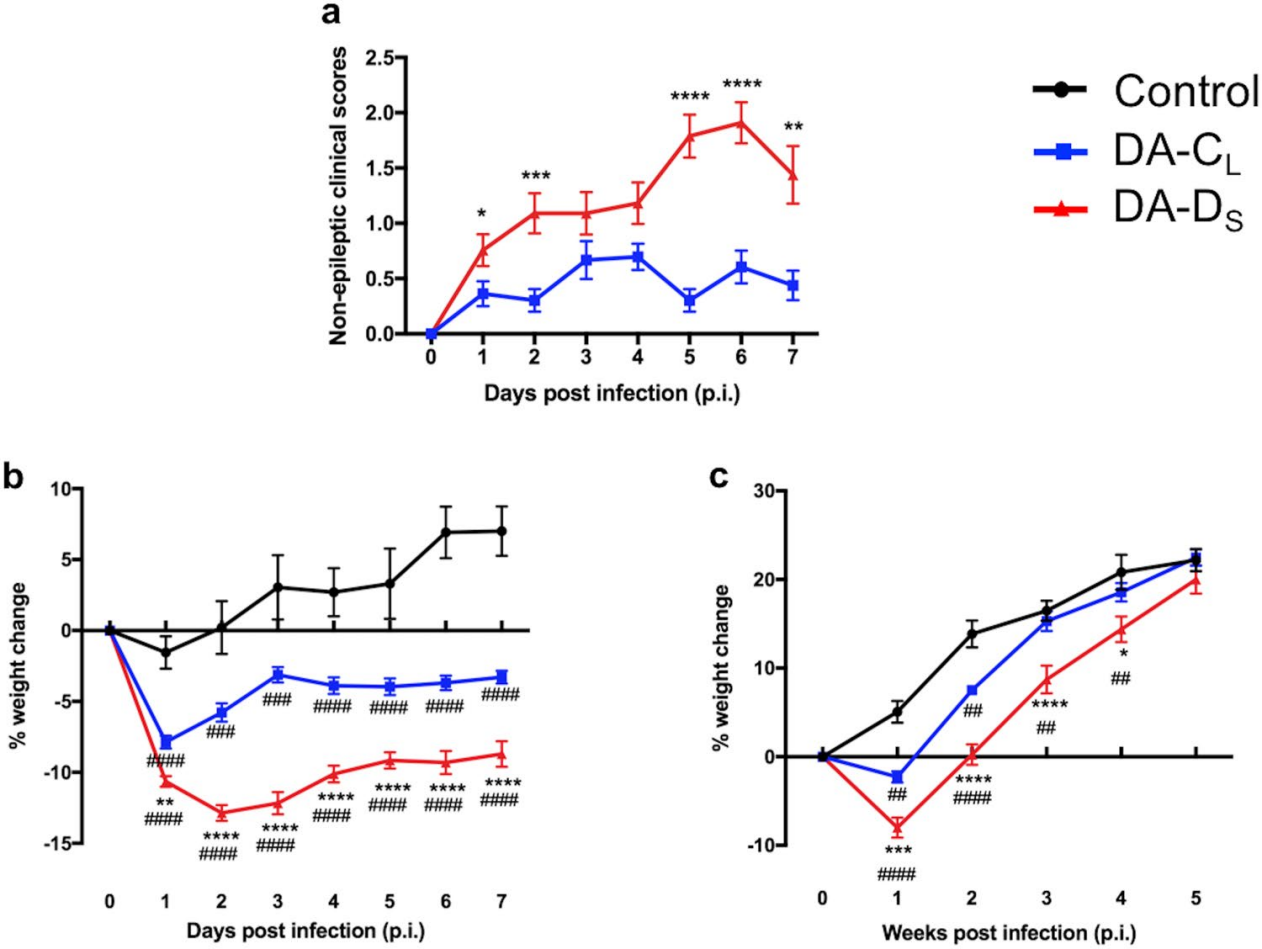
Mice infected with DA-D_S_ plaque variant displayed severe non-epileptic clinical signs and weight loss. (**a**) Mice were observed daily as described in Materials and Methods for signs of non-epileptic clinical disease during the acute phase (*p < 0.05, **p < 0.01, ***p < 0.001, ****p < 0.0001 by Mann-Whitney test). Mice from the control and infected groups were weighed (**b**) daily until day 7 p.i. and then (**c**) once weekly until week 5 p.i. (*p < 0.05, **/^##^p < 0.01, ***/^###^p < 0.001, ****/^####^p < 0.0001 by Repeated Measures two-way ANOVA with Tukey’s multiple comparisons test). Graphs (**a**,**b**) show pooled results from four separate experiments expressed as mean ± SEM, N = 4 in control group and N = 32–33 per infected group. Graph (**c**) shows pooled results from two separate experiments expressed as mean ± SEM, N = 4 in control group and N = 13 per infected group.

We evaluated the effects of plaque-variant infection on non-epileptic clinical signs during the acute phase. Most mice developed hunched backs, ruffled fur, and/or ataxia post-infection. The overall mean clinical score for DA-D_S_-infected group was 1.32 ± 0.16, whereas DA-C_L_-infected group scored 0.48 ± 0.06. Thus, encephalitis-like signs were more prominent among DA-D_S_-infected mice compared to DA-C_L_-infected mice (Fig. 3a) (Mann-Whitney test; p < 0.05 on day 1 p.i., p < 0.001 on day 2 p.i., p > 0.05 on days 3, and 4 p.i., p < 0.0001 on days 5 and 6 p.i., and p < 0.01 on day 7 p.i.). Furthermore, four of the DA-D_S_- and two of the DA-C_L_-infected mice developed flaccid paralysis of hind limb(s) during the acute phase. DA-D_S_-infected mice that developed flaccid paralysis also exhibited seizures.

### Mice infected with DA-D_S_ plaque variant had a significant delay in their recovery

Weight is one parameter used to monitor the health of mice. Using repeated measures two-way ANOVA, we found very significant differences in weights of mice among groups that were influenced by infection with plaque variants (F(2,67) = 59.13, p < 0.0001), and days p.i. (F(7,469) = 47.32, p < 0.0001). The interaction between infection and days p.i. was also found to be significant (F(14,469) = 25.73, p < 0.0001). Further analysis showed that all mice lost weight following i.c. injection, but weight loss was less marked in the control group and highest in the DA-D_S_-infected group as illustrated in Fig. 3b. Control mice gained weight after day 2 p.i., while mice in the infected groups continued to lose weight during the acute phase of infection. (Tukey’s multiple comparisons post hoc test; for control vs. DA-C_L_ groups- p < 0.0001 on days 1, 4, 5, 6, 7 p.i., p < 0.001 on days 2 and 3 p.i.; for control vs. DA-D_S_ groups- p < 0.0001 on days 1 till 7 p.i.; for DA-C_L_ vs. DA-D_S_ groups- p < 0.01 on day 1 p.i. and p < 0.0001 on days 2 till 7 p.i.).

We conducted repeated measures two-way ANOVA, and found that both infection with plaque variants (F(2,27) = 15.12, p < 0.0001) and days p.i. (F(5,135) = 246.4, p < 0.0001) significantly affected weights of mice during the chronic phase. The interaction between infection and days p.i. was also found to be significant (F(10,135) = 6.976, p < 0.0001). The DA-C_L_-infected mice began to gain weight during week 2 p.i., and were equal in weight to control mice by week 3 p.i. DA-D_S_-infected mice started gaining weight during week 3 p.i., but their weights remained significantly lower than the other groups until week 4 p.i. (Fig. 3c). (Tukey’s multiple comparisons post hoc test; for control vs. DA-C_L_ groups- p < 0.01 on weeks 1 and 2 p.i., p > 0.05 on weeks 3, 4, and 5 p.i.; for control vs. DA-D_S_ groups- p < 0.0001 on weeks 1 and 2 p.i., p < 0.01 on weeks 3 and 4 p.i., and p > 0.05 on week 5 p.i.; for DA-C_L_ vs. DA-D_S_ groups- p < 0.001 on week 1 p.i., p < 0.0001 on weeks 2 and 3 p.i., p < 0.05 on week 4 p.i., and p > 0.05 on week 5 p.i.). Thus, compared to other groups, recovery in DA-D_S_-infected group was significantly delayed.

### Increased viral burden in mice infected with DA-D_S_ plaque-variant

**Figure 4:**
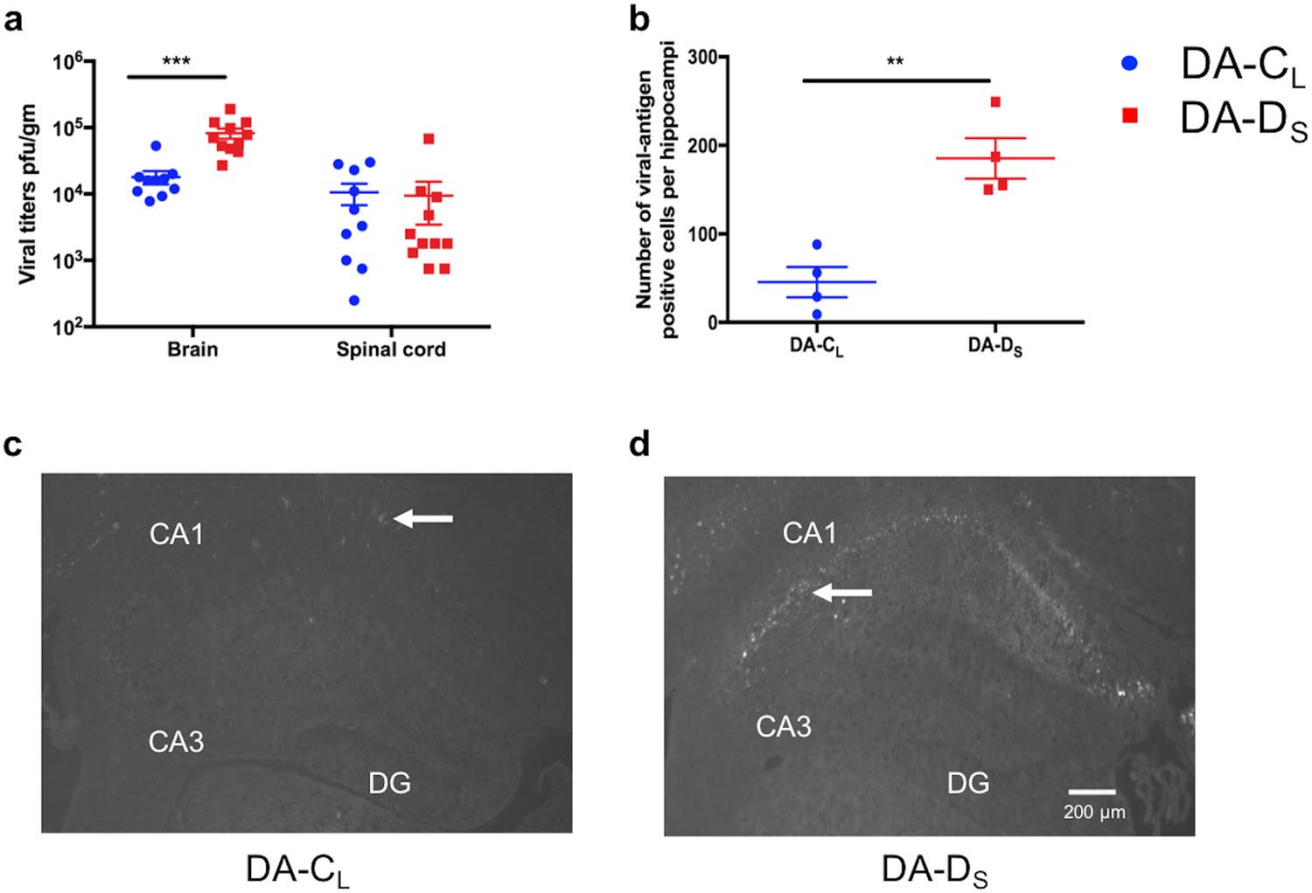
Increased viral burden in mice infected with DA-D_S_ plaque-variant. (**a**) Using plaque assay, viral burden was determined in brain and spinal cord homogenates from day 7 p.i. (**b**) Viral-antigen positive cells were detected in the hippocampus using rabbit anti-TMEV at day 7 p.i. The number of viral-antigen positive cells in the left and right hippocampi was enumerated and compared between the infected groups. (**p < 0.01, ***p < 0.001 by t test with Welch’s correction). (**c**,**d**) The ⇦ indicates viral-antigen positive cells in the CA1 and CA2 pyramidal layers of the hippocampus. Some of the cortical neurons surrounding the CA1 and CA2 pyramidal layers also stained positive for viral-antigen. Graph (**a**) shows mean ± SEM, N = 10 in DA-C_L_ group and N = 11 in DA-D_S_ group. Graph (**b**) shows mean ± SEM, N = 4/infected group. Cornu ammonis1 (CA1), cornu ammonis3 (CA3), and dentate gyrus (DG).

We assessed viral burden in brain and spinal cord homogenates from day 7 p.i. As shown in Fig. 4a, viral titers in the brains from DA-D_S_-infected mice (Mean ± SEM = 82273 ± 14170 pfu/g) were approximately five-fold higher than in DA-C_L_-infected mice (Mean ± SEM = 18010 ± 4086 pfu/g) (t test with Welch’s correction; t(11.64) = 4.36, p = 0.0010). However, viral titers in the spinal cords were found to be similar between the infected groups, with mean titers from DA-D_S_-infected mice being 9409 ± 5950 pfu/g, and from DA-C_L_-infected mice were 10560 ± 3758 pfu/g (t test with Welch’s correction; t(16.63) = 0.16, p > 0.05).

### Increased number of viral-antigen positive cells in the hippocampus of DA-D_S_-infected mice

We analyzed coronal sections of mouse brain from the infected groups for virus localization in the hippocampus. At day 7 p.i., the number of virus-antigen positive cells in the hippocampi were significantly higher in DA-D_S_-infected mice (Mean ± SEM = 185.3 ± 22.78) compared to DA-C_L_-infected mice (Mean ± SEM = 45.50 ± 17.13) (Fig. 4b) (t test with Welch’s correction; t(5.57) = 4.90, p < 0.01). Viral-antigen was mainly present in the CA1 and CA2 pyramidal layers, while it was completely absent from the CA3 pyramidal layer and the granule cell layer of the dentate gyrus. In addition to virus-antigen positive cells in the hippocampus, viral infection was noted in the cortex. But in both the infected groups, virus-antigen spread was limited to the cortical area nearest to the CA1 and CA2 hippocampal regions (Fig. 4c,d).

### Increased glial pathology among DA-D_S_ infected mice

**Figure 5:**
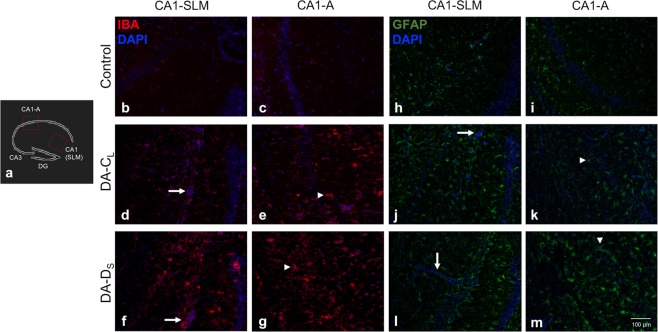
Increased glial pathology among DA-D_S_ infected mice. Microglia/macrophages were detected with an antibody against Iba1, astrocytes with an antibody against Gfap and counter stained with Hoechst. (**a**) Schematic diagram of the coronal section of hippocampus depicting different areas of CA1 field. (**b**,**c**) Images of corresponding regions of hippocampus from control group depicting Iba1+ cells, and (**h**,**i**) images depicting Gfap+ cells. In comparison to control, (**d**–**g**) activated macrophage/microglia and (**j**–**m**) astrocytes were found in CA1 region of hippocampus in both DA-C_L_- and DA-D_S_-infected groups. The ⇦ indicates Iba1+ or Gfap+ cells lining PVCs, and the Δ indicates activated cells. Cornu ammonis1 (CA1), cornu ammonis3 (CA3), dentate gyrus (DG), stratum lacunosum-moleculare (SLM), and peri-vascular cuffs (PVCs).

Gliosis is a characteristic finding in several neurological diseases including epilepsy^26–28^. At day 7 p.i., we examined brain sections from infected mice for the expression of Iba1+ microglia/macrophages, and the number and morphology of Gfap+ astrocytes in the hippocampus. Unlike the presence of resting microglia (ramified phenotype) in control mice (Fig. 5b,c), microglia/macrophages were found to be highly activated (amoeboid phenotype) in the infected groups (Fig. 5d–g). However, we found approximately a two-fold increase in the Iba1+ expression in DA-D_S_-infected mice (Mean ± SEM = 2.037 ± 0.28) compared to that in DA-C_L_-infected mice (Mean ± SEM = 1 ± 0) (t test; t(4) = 3.62, p < 0.05). This effect was more pronounced in the CA1-CA2 regions of hippocampus, where macrophages/microglia were found to encroach upon the pyramidal neuronal layer.

In general, we found an increase in the number of astrocytes in brain sections from DA-D_S_-infected mice (Mean ± SEM = 1169 ± 113) compared to that in DA-C_L_-infected mice (Mean ± SEM = 900.9 ± 120.8). Nonetheless, the astrocyte count was not significantly different between the infected groups (t test with Welch’s correction; t(5.97) = 1.62, p > 0.05). This could be because of the small sample size and/or pooled results from seized and non-seized mice in the DA-C_L_-infected group. Interestingly, the non-seized mice in DA-C_L_-infected group had fewer astrocytes compared to the seized mice in the DA-D_S_ and DA-C_L_-infected groups.

Compared to the non-reactive/resting astrocytes (Fig. 5h,i), reactive astrocytes exhibit increased GFAP expression and hypertrophy of the cell body and cellular processes^27^. In our study, astrocytes in the hippocampus showed variability in the extent of GFAP expression and hypertrophy between and within the infected groups. The morphology of astrocytes varied from mildly activated to highly activated among the DA-C_L_-infected mice (Fig. 5j,k), while most astrocytes from the DA-D_S_-infected mice were highly activated and hypertrophied (Fig. 5l,m).

### Extensive inflammation and neuronal damage in the hippocampus of mice infected with DA-D_S_ variant

**Figure 6:**
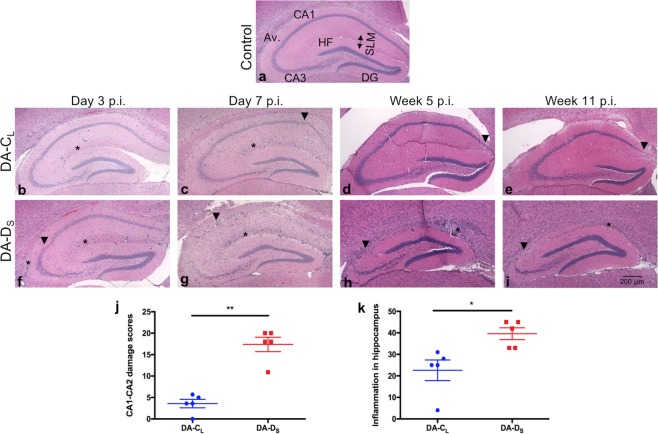
Extensive inflammation and neuronal damage in the hippocampus of mice infected with DA-D_S_ variant. (**a**) H&E stained coronal section of hippocampus from control mice. Hippocampal regions from the infected groups were analyzed at days 3 (**b**,**f**) and 7 (**c**,**g**) and weeks 5 (**d**,**h**) and 11 (**e**,**i**) p.i. for histopathology. (**b**–**e**) are the representative images from DA-C_L_-infected group and (**f**–**i**) are the representative images from DA-D_S_-infected group. At day 7 p.i., the percent loss of CA1-CA2 neurons was estimated and ranked on a scale of 0 to 10. (**c**) A representative section of hippocampus from DA-C_L_-infected group presenting CA1-CA2 damage score 2. (**g**) A representative section of hippocampus from DA-D_S_-infected group presenting CA1-CA2 damage score 10. (**j**) The damage to the CA1 and CA2 pyramidal layers (**p < 0.01 by Mann-Whitney test) and (**k**) the intensity of inflammation as determined by the number of perivascular cuffs and inflammatory foci (*p < 0.05 by t test with Welch’s correction) were compared between the infected groups at day 7 p.i. The ▼ indicates neuronal loss and the *indicates inflammation in the images. Graphs show mean ± SEM, N = 5 per infected group. Cornu ammonis1 (CA1), cornu ammonis2 (CA2), cornu ammonis3 (CA3), dentate gyrus (DG), alveus (Av.), hippocampal fissure (HF) and stratum lacunosum-moleculare (SLM).

To evaluate the effects of DA plaque-variants on pathology in the hippocampus, we examined H&E stained coronal sections of brain for inflammation and neuronal damage at various time points. The preliminary data from day 3 p.i. showed that compared to controls (Fig. 6a), infected mice developed inflammation (perivascular cuffs [PVCs] and inflammatory foci) at the alveus, hippocampal fissure and stratum lacunosum-moleculare (SLM) area of CA1 and CA2 regions, while only the DA-D_S_-infected mice showed neuronal loss at CA1 and CA2 pyramidal layers (Fig. 6b,f). By day 7 p.i., the neuronal damage was also found in the DA-C_L_-infected group along with the existent inflammation (Fig. 6c). In contrast, there was a marked increment in the intensity of inflammation and CA1 and CA2 neuronal loss in the DA-D_S_-infected group (Fig. 6g). The extent of acute neuronal loss (Mann-Whitney test; p < 0.01) was significantly greater following DA-D_S_-infection compared to DA-C_L_-infection (Fig. 6j). The extent of neuro-inflammation was also significantly higher for the DA-D_S_-infected group (Mean ± SEM = 39.60 ± 2.75) than for the DA-C_L_-infected group (Mean ± SEM = 22.60 ± 4.78) (t test with Welch’s correction; t(6.39) = 3.08, p = 0.0199) (Fig. 6k). In the chronic phase, the inflammation mostly subsided and no further neuronal damage was found in the DA-C_L_-infected mice (Fig. 6d,e), while the inflammation was exacerbated in the DA-D_S_-infected mice by week 5 p.i. (Fig. 6h) and persisted at week 11p.i., which was the latest time-point at which histological analysis was performed (Fig. 6i). Moreover, we did not find any damage to the dentate gyrus and CA3 neurons in either of the infected groups, which is consistent with other studies using the DA strain^22^.

Our results show that both plaque variants are capable of inducing inflammation in the hippocampus, but the intensity of inflammation is much more severe following DA-D_S_-infection compared to DA-C_L_-infection. In the chronic phase, inflammation resolves following DA-C_L_-infection, while it continues to increase following DA-D_S_-infection. The DA-D_S_ variant also induces an early and severe onset of neuronal damage and almost complete loss of their CA1 and CA2 neurons by day 7 p.i. In contrast, the DA-C_L_ variant causes minimal neuronal loss in the acute phase, which does not progress in the chronic phase.

### Acute inflammation was more pronounced at the lumbar segment of spinal cord following DA-D_S_-infection

Unlike SJL mice, infection with the DA virus fails to cause demyelinating lesions in the spinal cord of C57BL/6 mice^11^. To study the impact of DA plaque variants on spinal cord disease in C57BL/6 mice, we analyzed H&E stained spinal cord sections for damage at acute (days 3 and 7 p.i.) and chronic (weeks 5 and 11 p.i.) phases of infection.

At day 3 p.i., no significant lesions were observed in the spinal cords of any of the infected mice in comparison to control mice. By day 7 p.i., both the infected groups developed meningitis, PVCs, inflammatory foci, and/or possible neuronal degeneration at the ventral and/or lateral horns of the gray matter of spinal cord. In some mice, inflammation even extended to the dorsal horn of the gray matter, and ventral and lateral columns of the white matter.

The magnitude of inflammation was similar at the cervical and thoracic segments between the infected groups, but the lumbar and sacral segments of the DA-D_S_-infected group showed more neuropathology than the DA-C_L_-infected group. In the DA-D_S_-infected group, lesions were found in 6/6 mice at the lumbar segment and 4/6 mice at the sacral segment, while in the DA-C_L_-infected group, lesions were only found in 2/5 mice at the lumbar and sacral segments (Supplementary Fig. S1).

Upon comparing the cumulative distribution of the scores, we found that DA-D_S_ plaque variant induced significantly more lesions at the lumbar segment in comparison to the DA-C_L_ plaque variant (Supplementary Fig. S1) (Kolmogorov-Smirnov test; for cervical and thoracic segments-D = 0.13, p > 0.05; for lumbar segment-D = 0.80, p < 0.05; and for sacral region-D = 0.47, p > 0.05).

By week 5 p.i., the inflammation subsided in both the infected groups. In addition, we did not find demyelinating lesions in any of the infected mice at week 5 or 11 p.i. Our results show that although DA-C_L_-and DA-D_S_ plaque variants induce acute inflammation in the spinal cord, they fail to cause chronic demyelinating disease in C57BL/6 mice. Nonetheless, the deleterious effects of DA-D_S_ plaque variant were more prominent than that of DA-C_L_ plaque variant, specifically at the lumbar segment of the spinal cord.

### Increased immune cell infiltration into the CNS of mice following DA-D_S_-infection

**Figure 7:**
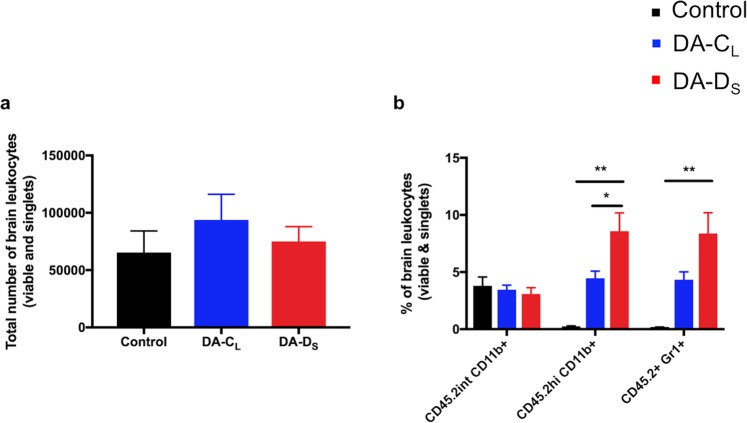
Increased immune cell infiltration into the CNS of mice following DA-D_S_-infection. As described in Materials and Methods, brain leukocytes from control and infected mice were collected at day 3 p.i. using Percoll gradient, stained for cell specific markers, and analyzed by flow cytometry. (**a**) The total number of brain leukocytes collected. (**b**) % CD45.2int CD11b+, % CD45.2hi CD11b+, and % CD45.2+ Gr1+ cells of the total brain leukocytes. Cells were gated based upon their CD45.2int/low CD11b+ or CD45.2+ Gr1+ expression. Graphs show pooled results from four separate experiments expressed as mean ± SEM, N = 5 in control group and N = 13 per infected group. (*p < 0.05, **p < 0.01 by one-way ANOVA with Tukey’s multiple comparisons test). Intermediate (int) and high (hi).

TMEV infection stimulates the activation of resident immune cells and infiltration of peripheral immune cells into the CNS^22,25,29^. To immuno-phenotype leukocyte populations, we harvested brain leukocytes from control and infected mice at day 3 p.i., as seizures peak at this time point. The total number of brain leukocytes collected (Fig. 7a) was similar among all the groups (One-way ANOVA; F(2,28) = 0.48, p > 0.05). To differentiate between microglia and macrophage/monocyte/neutrophil populations, we gated cells based on their CD45.2 and CD11b expression. As depicted in Fig. 7b, we did not find any significant differences in the expression levels of CD45.2int CD11b+ cells (microglia) among the groups (One-way ANOVA; F(2,28) = 0.36, p > 0.05), however, the expression levels of CD45.2hi CD11b+ cells (macrophages/monocytes/neutrophils) were found significantly different (F(2,28) = 8.49, p = 0.0013). Interestingly, the expression levels of CD45.2hi CD11b+ cells in the DA-D_S_-infected group (Mean ± SEM = 8.59 ± 1.59) were found significantly higher than that in control (Mean ± SEM = 0.24 ± 0.05) or DA-C_L_ group (Mean ± SEM = 4.46 ± 0.62) (Tukey’s multiple comparisons post hoc test; control vs. DA-D_S_ group-p = 0.0014; control vs. DA-C_L_ group-p > 0.05; DA-C_L_ vs. DA-D_S_ group-p < 0.05). The expression levels of CD45.2+ Gr1+ cells were also significantly different among the groups (One-way ANOVA; F(2,28) = 6.44, p < 0.01). There was a significant increase in the expression levels of CD45.2+ Gr1+ cells in the DA-D_S_-infected group (Mean ± SEM = 8.39 ± 1.80) compared to the control group (Mean ± SEM = 0.16 ± 0.04), but not the DA-C_L_ group (Mean ± SEM = 4.33 ± 0.68) (Tukey’s multiple comparisons post hoc test; control vs. DA-D_S_ group-p < 0.01; control vs. DA-C_L_ group-p > 0.05; DA-C_L_ vs. DA-D_S_ group-p > 0.05).

Our results indicate that DA-D_S_ plaque-variant had a significant impact on the infiltration of peripheral innate immune cells into the CNS. On one hand, the inflammatory response is crucial for protection against virus infection, but on the other hand, the dysregulated inflammation initiates widespread hippocampal damage, leading to seizure development.

### Genetic Differences between viral strains

Total RNA was extracted from DA-C_L_ and DA-D_S_ infected L2 cells and cDNAs representing overlapping amplicons spanning nucleotides (nts) 137-3′poly(A) tail (nt positions are all relative to Genbank Accession M20301)^30^ were synthesized by RT-PCR, purified by agarose gel electrophoresis and sequenced as described in Materials and Methods. Sequences were deposited in Genbank as accessions MK343442 (DA-C_L_) and MK343443 (DA-D_S_). A list of the overlapping amplicons can be found in Supplementary Table S2. The nucleotide sequences of the DA-C_L_ and DA-D_S_ variants and the translated proteins were aligned and compared to each other and to the three other TMEV-DA complete genomes in Genbank (Genbank Accessions M20301, JX443418, and KF680264)^30,31^ with Clustal Omega. Sequence differences are summarized in Table 1. There are three nucleotide differences between DA-C_L_ and DA-D_S_. The first is a T1244C change in DA-C_L_ compared to both DA-D_S_ and the three complete genome sequences for the DA strain of TMEV that are present in Genbank. This change results in a M60T amino acid change in the DA-C_L_ leader protein and a W56R change in the L* protein relative to both DA-D_S_ and the translations of the three TMEV-DA sequences in Genbank. The second is in the coding sequence for the helicase domain of protein 2C, where DA-D_S_ contains a T5030C change relative to DA-C_L_ and the Genbank sequences. This produces a corresponding I133T mutation in the DA-D_S_ 2C protein relative to the 2C protein encoded in DA-C_L_ and the other three TMEV-DA sequences in Genbank. The third nucleotide difference between the DA-C_L_ and DA-D_S_ variants is coding silent at position 6054, where DA-D_S_ and the three other viruses we analyzed contain an A at this position and the DA-C_L_ virus contains a G.

**Table 1:** Summary of sequence differences between DA-C_L_ and DA-D_S_ and Genbank Accessions M20301, JX443418, and KF680264.

Nucleotide Position	DA-D_S_	DA-D_S_ amino acid	DA-C_L_	DA-C_L_ amino acid	Genbank M20301	Genbank M20301 amino acid	Genbank JX443418	Genbank JX443418 amino acid	Genbank KF680264	Genbank KF680264 amino acid
475	C	NA	C	NA	T	NA	T	NA	T	NA
1244	T	L protein 60M L*protein 56W	C	L protein 60T L*protein 56R	T	L protein 60M L*protein 56W	T	L protein 60M L*protein 56W	T	L protein 60M L*protein 56W
1929	C	VP2 protein 141K	C	VP2 protein 141K	G	VP2 protein 141K	T	VP2 protein 141N	G	VP2 protein 141K
5030	C	2C protein 133T	T	2C protein 133I	T	2C protein 133I	T	2C protein 133I	T	2C protein 133I
6054	A	3C protein 40Q	G	3C protein 40Q	A	3C protein 40Q	A	3C protein 40Q	A	3C protein 40Q
7813	G	3D protein A410	G	3D protein A410	C	3D protein A410	C	3D protein A410	C	3D protein A410

There are three additional sites where the DA-C_L_ and DA-D_S_ sequences differ from all of the complete genomes in Genbank. In the 5′ untranslated region (5′UTR) there is a C at position 475 in the DA-C_L_ and DA-D_S_ sequences and a T is present in all three TMEV genome sequences in Genbank. Nucleotide 7813 is a G in both DA-C_L_ and DA-D_S_ whereas all three of the Genbank Accessions contain a C at this position. This difference results in an alanine at amino acid 410 in the DA-C_L_ and DA-D_S_ 3D proteins, whereas the other 3 viruses encode a proline at this position. A third difference is present at nucleotide 1929 where both DA-C_L_ and DA-D_S_ contain a C, whereas a T is present in Genbank Accession JX443418 and a G is present in Genbank Accessions M20301 and KF680264. This nucleotide difference is coding silent relative to the translation of M20301 and KF680264 and is thus unlikely to be significant. There are approximately 40 other sites where the DA-C_L_ and DA-D_S_ sequences are discordant from one or two of the three complete TMEV-DA genome sequences but are identical to at least one TMEV-DA genome sequence in Genbank (not shown). The vast majority of these correspond to coding silent polymorphisms amongst the three TMEV genomes in Genbank.

## Discussion

Fujinami and co-workers were the first to characterize the TMEV-induced epilepsy model. They found that C57BL/6 mice infected with the DA strain of TMEV exhibit symptomatic seizures in the acute phase, recover from viral infection, but after an unspecified latent phase develop unprovoked recurrent seizures (epilepsy). In this infection-driven model, the lesions are mainly concentrated in the CA1 and CA2 regions of the hippocampus. In the chronic phase, the neuronal loss and gliosis progresses to cause hippocampal atrophy and glial scarring, termed hippocampal sclerosis (HS)^13,19,32–34^. HS is also the hallmark neuropathology of mesial temporal lobe epilepsy (MTLE), the most prevalent form of human epilepsy^35–37^. Since DA-infection shares similarities with MTLE, it serves as an excellent model to study epileptogenesis and epilepsy.

Our current work investigated the neuropathogenesis of large (DA-C_L_) and small (DA-D_S_) plaque-size variants of DA strain of TMEV-induced seizures. The *in vivo* studies revealed a striking contrast between the phenotypes of the DA-C_L_ and DA-D_S_ plaque variants. We found that the percentage of seized mice was significantly higher following DA-D_S_-infection (~94%) compared to DA-C_L_-infection (~12%). We recorded repeated seizures in the DA-D_S_-infected group that began on day 2 and ceased after day 7 p.i., whereas seizures in the DA-C_L_-infected group were only observed on day 3 p.i. The frequency and severity (Racine stage 4 or 5) of seizures in the DA-D_S_-infected group peaked on day 3 p.i. and remained high until day 5 p.i. In a previous study, where similar titers (2.5 × 10^5^ pfu) of the DA strain were used and mice were examined twice daily, acute seizures were observed in ~57% of the infected C57BL/6 mice^38^. The phenotypic variability between the DA strain and the plaque variants indicate that the neuro-virulence is DA-D_S_-plaque variant > DA strain > DA-C_L_-plaque variant.

Furthermore, we monitored clinical illness including weight loss and non-epileptic clinical signs in mice following infection. Compared to DA-C_L_, the DA-D_S_ plaque variant caused severe illness (ruffling, hunched back and ataxia) in mice during the acute phase. In addition, the acute weight loss was highest in the DA-D_S_-infected mice, and their weights remained significantly lower than the control or DA-C_L_-infected mice until one-month p.i. This implies that there was significant delay in the recovery among DA-D_S_-infected mice.

Viral titers remained significantly higher in the brains of DA-D_S_- infected mice compared to those of DA-C_L_-infected mice at day 7 p.i. Similar to the DA infection^22^, we also detected virus-antigen positive cells in the hippocampus at day 7 p.i., localized in the CA1 and CA2 pyramidal neurons, but the neurons in dentate gyrus and CA3 region were not affected. The number of viral-antigen positive cells was significantly higher in the DA-D_S_-infected group compared to the DA-C_L_-infected group. These findings confirm that DA-D_S_-, but not DA-C_L_-, plaque variant could efficiently replicate *in vivo*. Since viral-antigen positive cells were mainly confined to the CA1 and CA2 pyramidal layers of the hippocampus, we examined these regions for neuronal loss. By day 7 p.i., the DA-D_S_-infected group had wide-spread loss of CA1 and CA2 neurons, and increased presence of PVCs and inflammatory foci in the hippocampal fissure and near CA1 and CA2 pyramidal layers. In contrast, neuronal loss in the DA-C_L_-infected group was patchy, with significantly fewer PVCs and inflammatory foci. In the chronic phase, mice from the DA-D_S_-infected group developed evident anatomical changes at CA1 and CA2 regions of hippocampus. The extensive loss of pyramidal neurons in the DA-D_S_-infected group may have emanated from the high viral burden, inflammation, and/or seizures from the acute phase.

Moreover, we detected viral burden and inflammation in the spinal cords of infected mice at day 7 p.i. The magnitude of inflammatory lesions varied within the spinal cord segments of the same mouse and between the infected mice. However, the most marked lesions were found in the lumbar segment of the spinal cord among DA-D_S_-infected mice. Mice also developed ataxia/flaccid paralysis of the hind limb(s) during the first week, which was more pronounced following DA-D_S_-infection. Therefore, we suggest that mice develop short-lived hind limb deficits, the intensity of which depends upon the severity of spinal cord lesions. Nonetheless, the intensity and duration of inflammation and viral burden was much higher in the brains than the spinal cords of the infected mice.

TMEV-infection triggers a significant host immune response, including the activation of resident (glia) and the infiltration of peripheral immune cells into the CNS. The immune response is crucial for protecting the CNS against viral infection, but also leads to massive neuronal destruction in the process. The neuronal damage (particularly of CA1 and CA2 pyramidal neurons) and seizures begin very early following TMEV (DA strain)-infection (day 2–3 p.i.), a time at which the adaptive immune response is not significant, implying that these events are mainly mediated by innate immune responses^22,25,29,34^. Here, we found that at day 3 p.i, the peak time for seizures in our study, the percentage of CD45.2hi CD11b+ cells (infiltrating innate immune cells), but not CD45.2int CD11b+ cells (microglia), were significantly higher in the DA-D_S_-infected group compared to the control and DA-C_L_-infected groups. At day 7 p.i., we found a significant increase in the expression of Iba1+ macrophages/microglia in the hippocampus following DA-D_S_ infection than DA-C_L_ infection. In addition, infected mice displayed reactive astrocytes, and seized mice had higher number of astrocytes than the non-seized mice. Thus, the increased levels of infiltrating innate immune cells and reactive gliosis are detrimental to the hippocampal neurons and result in seizures following DA-D_S_-infection.

Cardioviruses such as TMEV translate their genomes as a polyprotein precursor that for TMEV-DA is 2301 amino acids long. This polyprotein subsequently undergoes autoproteolytic cleavage to yield 12 viral proteins, one of which is the 76 amino acid leader protein^39–42^. The demyelinating strains of TMEV such as TMEV-DA also express an 18-kDA L* protein from an alternate open reading frame first identified by Kong and Roos^43^. The leader protein contains an N-terminal Zinc binding Cys-His-Cys-Cys (C-H-C-C) motif essential for this protein’s ability to inhibit IFN-α/β production, allowing for viral persistence^31,44–47^. Part of the mechanism for this inhibition of interferon induction has been linked to leader protein binding to nucleoporins and blocking mRNA nuclear export^48,49^. The Leader protein also contains an acidic domain, a serine/threonine rich domain, which in TMEV contains two phosphorylation sites, and a C-terminal TMEV-specific domain that plays a role in cellular localization and trafficking between the nucleus and cytoplasm^49,50^. L* is synthesized from an overlapping ORF 13 nucleotides downstream from the AUG start codon for the polyprotein^47,51^ and has been shown to inhibit the interferon-inducible OAS/RNase L pathway allowing for the evasion of host innate immunity^31^. The ability to impair the innate host immune response in certain cell types by either Leader or L* contributes to viral evasion of the host immune response and viral persistence^44,48,49,51,52^. Mutations in leader result in significantly less neurovirulence as indicated by decreased viral persistence in the CNS^46^. Our data show that the thymine to cytosine mutation at position 1244 in DA-C_L_ compared to both DA-D_S_ and the three TMEV Genbank sequences results in a methionine to threonine amino acid change at position 60 in the DA-C_L_ leader protein and a tryptophan to arginine change at position 56 for the L* protein. Interestingly, mutation of Leader methionine 60 to valine, leucine, or isoleucine has been shown to block binding of leader protein to nucleoporins, resulting in less inhibition of RNA export from the nucleus, and thus increased interferon induction *in vitro*, and decreased viral persistence *in vivo*^47,49^. Thus, we postulate that these mutations in the Leader and L* proteins of DA-C_L_ lead to the attenuating phenotype of the DA-C_L_ variant in the C57BL/6 mouse model of epilepsy. Compared to the DA-D_S_ variant, infection with the DA-C_L_ variant resulted in less viral burden and less neuro-inflammatory burden in the hippocampus of our DA-C_L_ infected mice. The overall result was better recovery of mice infected with the DA-C_L_ variant versus the DA-D_S_ and significantly fewer and less severe episodes of seizure activity in DA-C_L_ infected mice. Our data are consistent with previous findings that mutations in the leader protein^44^ and the L* protein^39,49^ both reduce viral neurovirulence and virus persistence in macrophages *in vitro*, and in the brain and spinal cord in mice^39,52,53^. Experiments are ongoing to elucidate the relative importance of the mutation in leader versus L*, as current research has yielded somewhat controversial claims as to the importance of each protein in viral persistence^31,39^. The sequence differences between DA-C_L_ and DA-D_S_ at nucleotides 5030 and 6054, respectively, appear to be less likely to contribute to the attenuation of DA-C_L_. The first because the nucleotide at this position in DA-C_L_ is identical to that in the other three TMEV stains in Genbank, and the latter because it is coding silent. It is possible however that the sequence difference between DA-D_S_ and the Genbank and DA-C_L_ sequences accounts for the small plaque phenotype of DA-D_S_.

The H-2D MHC class I locus strongly influences the susceptibility of the inbred mouse strains to the demyelinating disease or epilepsy. C57BL/6 mouse strain (*H-2*^*b*^ haplotype) is resistant to the demyelinating disease but susceptible to epilepsy, whereas the SJL mouse strain (*H-2*^*s*^ haplotype) is susceptible to the demyelinating disease but do not develop epilepsy^23,54–56^. Our findings in C57BL/6 mice together with the previous study by Oleszak *et al*. in SJL mice^21^ corroborate that both the C57BL/6 and SJL mouse strains develop severe neuro-pathological lesions, clinical disease, and viral burden following DA-D_S_-infection, while the magnitude of the clinical disease in both the mouse strains is minimal following DA-C_L_-infection. Hence, the neuro-virulence of DA-D_S_ and DA-C_L_ plaque-variants is independent of the H-2D MHC class I locus in C57BL/6 and SJL mouse strains.

Our results are in contrast to the recent paper published on two naturally occurring variants of the BeAn strain of TMEV, (BeAn-1 and BeAn-2). They found that BeAn-1 was neuro-virulent in only the SJL mouse strain causing demyelinating disease (MS), while BeAn-2 was neuro-virulent in only the C57BL/6 mouse strain causing acute seizures. Thus, in this particular instance, the neuro-virulence of the BeAn variants, unlike the DA variants we have studied, depends upon the genotype of C57BL/6 and SJL mice. The authors did not investigate if the BeAn variants formed different sized-plaques as the DA variants do *in vitro*^57^.

To conclude, we found that the DA-D_S_ plaque variant was extremely neuro-virulent and caused marked structural and functional alterations in the hippocampus of C57BL/6 mice, while the DA-C_L_ plaque variant had attenuated neuro-virulence. The previous study from SJL mice^21^ along with our current study in C57BL/6 mice indicate that the neuro-virulence of DA-D_S_ plaque variant is significantly higher than the DA-C_L_ plaque variant in the viral-induced models of two distinct neurological diseases, MS and epilepsy.

## Materials and Methods

### Mice

Three-week-old female C57BL/6 mice were purchased from Envigo Laboratories (Indianapolis, IN). All animal experiments (Fig. 1) were conducted in accordance with the protocols approved by the Institutional Animal Care and Use Committee (IACUC) of the Comparative Medicine Program at Texas A&M University.

### Virus

The DA-C_L_ (large) and DA-D_S_ (small) plaque variants of the DA strain of TMEV have been described previously^21^. Viruses were propagated and titered by plaque assay as described previously^18^.

### Infection

Mice were placed into 3 groups according to the virus used for infection: mock-infected controls, DA-C_L_, and DA-D_S_. Mice were housed in groups of 4–5 (cage dimensions: length 28 cm, height 12 cm and width 17.5 cm). At 4 weeks of age, mice in the infected groups were injected into the right mid-parietal cortex at a depth of approximately 1.5 mm with 2.0 × 10^5^ plaque forming units (pfu) of either DA-C_L_ or DA-D_S_ plaque variant in 20 μl of Dulbecco’s modified eagle medium (DMEM) (Sigma, Life Science, St. Louis, MO). Mice in the control group were injected with 20 μl of sterile 1x phosphate-buffered saline (PBS). All injections were performed under Isoflurane (IsoFlo, North Chicago, IL) anesthesia^58^.

### Body weight measurement

Mice were weighed daily until day 7 p.i. (acute phase), and then once a week until week 5 p.i. (chronic phase). Weight loss was calculated as percent of daily or weekly weights of mice compared to their weights at day 0 p.i.

### Clinical scores

For clinical scores, mice were observed twice daily, once in the morning and once during the evening for 1 hour each, until day 7 p.i. (acute phase). Additionally, two cages of infected mice (n = 9/infected group) were video recorded 24/7 and the resulting videos analyzed for acute seizures. The rater did not have prior knowledge of the subjects experimental conditions. Mice were scored for seizures based on the Racine scoring system; (1) Mouth and facial movements; (2) Head nodding; (3) Forelimb clonus; (4) Rearing; and (5) Rearing and falling progressing to tonic-clonic seizure^59^. For non-epileptic clinical signs, mice were scored as: score (0) no clinical signs; score (1) mildly ruffled, hunched, and/or ataxic; score (2) moderately ruffled, hunched, and/or ataxic; score (3) severely ruffled, hunched, and/or ataxic; score (4) paralysis; and score (5) moribund. Clinical evaluations were rated in a blinded manner.

### Tissue isolation

Mice were euthanized with 150 mg/kg beuthanasia-D special (Schering-Plough Animal Health Corp. Union, NJ) and perfused intracardially with 10 ml of sterile PBS. Tissues were harvested under sterile conditions and processed for individual experiments as described below.

### Plaque assay

At day 7 p.i., brains and spinal cords (n = 10 in DA-C_L_ group and n = 11 in DA-D_S_ group) were collected separately and clarified 10% w/v homogenates in DMEM were prepared and titrated by plaque assay as described previously^21,60^.

### Immunohistochemistry (IHC)

Brains (n = 3–4/infected group) were collected at day 7 p.i., post-fixed in 4% paraformaldehyde (PFA), and cryoprotected in 30% sucrose. Tissues were embedded in optimum cutting temperature (OCT) compound (Tissue-Tek 4583, Torrance, CA). 10-micron (μm) coronal sections were cut from OCT blocks. For each immunostain, four rostral-caudal matched sections containing dorsal hippocampi were analyzed per mouse.

Cryosections were incubated for 1 hour at 37 °C, hydrated, and blocked for 1 hour with 5% goat serum (16210-064; Gibco, Life Technologies, Grand Island, NY) and 0.1 or 0.3% Triton X-100 (9002-93-1; Sigma-Aldrich, St. Louis, MO) in PBS. Cryosections were incubated overnight at 4 °C with primary antibodies for microglia/macrophages, rabbit anti-ionized calcium-binding adapter molecule1 (Iba1)^61^ [1:200, 019–19741; Wako Chemicals USA, Inc., Richmond, VA], for astrocytes, chicken anti-glial fibrillary acidic protein(Gfap)^62^ [1:500, AB5541; EMD Millipore, Temecula, CA], or for TMEV-antigen positive cells, rabbit anti-TMEV^63^ [1:50]. The next day, cryosections were washed and then incubated for 1 hour with either Alexa Fluor 488 goat anti-chicken IgG [1:1000, A11039; Invitrogen, Life Technologies, Eugene, OR] or Alexa Fluor 594 goat anti-rabbit IgG [1:1000, A11037; Invitrogen, Life Technologies, Eugene, OR]. Cryosections were washed, counterstained with Hoechst 33342, trihydrochloride trihydrate [1:1000, H3570; Life Technologies, Eugene, OR] and mounted with Fluoromount-G (0100–01; SouthernBiotech, Birmingham, AL). For microglia/macrophages, the fluorescence intensity of Iba1 expression was determined in seven different but matched regions (20x) from left and right hippocampi each and totaled. For each image, the corrected total fluorescence was calculated as Integrated density- (area selected*mean fluorescence of backround readings). Changes in fold intensity was calculated by dividing the intensity of each DA-D_S_-infected mouse by the average intensity of DA-C_L_-infected mice. Activated astrocytes and TMEV-antigen positive cells were enumerated both in the left and right hippocampi and totaled. The analysis was done using ImageJ software (NIH).

### Histology: CNS

Brains and spinal cords were collected at days 3 and 7, and weeks 5 and 11 p.i., fixed in 10% formaldehyde, processed and embedded in paraffin wax. Four μm coronal sections of brain (containing dorsal hippocampi) and transverse sections of spinal cord from control (n = 3) and infected mice (n = 4–6/group) from acute (days 3 and 7 p.i.) and chronic (weeks 5 and 11 p.i.) phase were stained with H&E.

### Brain

Inflammation in hippocampus was assessed at day 7 p.i. by enumerating the number of inflammatory foci and PVCs in right and left hippocampi from each mouse. Previous studies have shown that acute neuronal loss in TMEV-infected C57BL/6 mice mainly occurs in the CA1 and CA2 regions of the hippocampus^22^. Therefore, we estimated the percent loss of CA1 and CA2 neurons and ranked it on a scale of 0 to 10. Scores from the left and right hippocampi were totaled, such that the highest possible cumulative score for each mouse was 20^64^. H&E stained brain sections from day 3 and weeks 5 and 11 p.i. were examined for the progression of neuropathology in the hippocampus.

### Spinal cord

The cervical, thoracic, lumbar and sacral segments of the spinal cords were examined for the presence of inflammatory foci, axonal/neuronal degeneration, and/or meningitis. Each spinal cord segment was graded separately on a scale of 0 to 4: score (0) the absence of any pathology; score (1) minimal pathological lesions (<10%); score (2) mild pathological lesions (10% to < 30%); score (3) moderate pathological lesions (30% to <70%); and score (4) severe pathological lesions (≥70%).

All images were acquired using a HRD076-NIK camera attached to OLYMPUS VANOX AHBS3 microscope, Spot software version: 5.2.21.17026.

### Flow cytometry

Whole brains (n = 5/control group and n = 13/infected group) were collected at day 3 p.i. in ice cold RPMI 1640 (Gibco, Life Technologies, Grand Island, NY), homogenized, filtered, and centrifuged at 500G for 5 min at room temperature. After discarding the supernatant, each pellet was suspended in 10 ml of 30% Percoll (17-0891-01; GE healthcare, Uppsala, Sweden) in PBS. This solution was gently overlaid onto 2 ml of 70% Percoll in PBS, and centrifuged 500G for 30 min at 18 °C. The leukocytes were collected from the interphase of the 30% and 70% Percoll layers, washed, and suspended in flow buffer containing 2% fetal bovine serum (FBS) [16000–044; Gibco, Invitrogen, Grand Island, NY] in PBS. Cells were treated with Anti-Mouse CD16/CD32 (1:100, 14-0161-82; eBioscience, San Diego, CA) for 10 min at 4 °C. For phenotyping, cells were stained with the anti-mouse antibodies as indicated below for 30 min at 4 °C, washed and fixed with 2% PFA prior to flow cytometric analysis on a Beckman Coulter MoFlo® Astrios™ High-Speed Cell Sorter. Data were analyzed using FlowJo^®^ software V10.0.8r1 (Mac OS X, FlowJo, LLC, Ashland, OR). CD45.2 was detected with clone 104 (1:100, 109805; BioLegend, San Diego, CA). CD11b was detected with clone M1/70 (1:500, 101224; BioLegend, San Diego, CA). Ly-6G (Gr-1) was detected with clone RB6-8C5 (1:500, 12-5931-82; eBioscience, San Diego, CA). Cell viability was assessed with Ghost dye Red 780 (1:100, 13–0865; Tonbo Biosciences, San Diego, CA). For compensation controls, UltraComp eBeads (01-2222-41; eBioscience, San Diego, CA) were used^65^.

### Sequencing of plaque variants

Tissue culture dishes (60 mm diameter) of confluent L2 cells were infected with DA-D_S_ or DA-C_L_ at an multiplicity of infection^66^ of 3 and incubated for 16 hours at 37 °C and total intracellular RNA extracted using the Omega Bio-Tek Total RNA Kit as per the manufacturer’s instructions. cDNA synthesis was performed with Superscript III (Invitrogen) using the primers listed in Supplementary Table S1. The resulting cDNAs were amplified by PCR to produce the overlapping amplicons listed in Supplementary Table S2. PCR was performed with ONETAQ DNA polymerase (New England Biolab) and the PCR primers listed in Supplementary Table S2. The most 3′ amplicon was amplified using the 3′RACE procedure^67^. The PCR conditions used for each amplicon were those recommended by the New England Biolab web tool (http://tmcalculator.neb.com/#!/)^68^. Amplified cDNAs were purified by 1% agarose gel electrophoresis, recovered from the gel using Bioline PCR and Gel Extraction kits, and the purified PCR products sequenced commercially (Eurofins) using the primers listed in Supplementary Table S3. Sequences were aligned using Sequencher (version 4.8) and a consensus sequence determined. The DA-C_L_ and DA-D_S_ sequences were aligned and compared for differences using L-align and ClustalOmega web platforms.

### Statistical analysis

Comparison of seizure duration within the group was analyzed using the one-way analysis of variance (ANOVA) with the post test for linear trend. Body weights were analyzed using the repeated measures two-way ANOVA with the Tukey’s multiple comparisons test. For the rest of the parametric analysis, the one-way ANOVA with the Tukey’s multiple comparisons test or two-tailed t-test with Welch’s correction was used. For non-parametric analysis, two-tailed Mann-Whitney test or Kolmogorov-Smirnov test was used. For nominal data (presence or absence of seizures), two-sided Fisher’s exact test was used. For all cases, significance was determined when p ≤ 0.05. Statistical analysis was done using GraphPad Prism version 6.0d (Mac OS X, GraphPad Software, La Jolla, CA).

## Supplementary information

Supplementary Information

## Data Availability

The datasets generated and analysed during the current study are available in Genbank as accessions MK343442 (DA-C_L_) and MK343443 (DA-D_S_).
