# Supplementary material for: Characterization of Plaque-Sized Variants of Daniel’s (DA) Strain in Theiler’s Virus-Induced Epilepsy
Source: Sci Rep. 2019 Mar 5;9:3444. doi: 10.1038/s41598-019-38967-z (PMC6401140; doi:10.1038/s41598-019-38967-z)
Supplement: Supplementary file 1 — Supplementary Information [file 41598_2019_38967_MOESM1_ESM.pdf]

CHARACTERIZATION OF PLAQUE-SIZED VARIANTS OF DANIEL'S (DA) STRAIN IN THEILER'S VIRUS-INDUCED EPILEPSY

M. Bijalwan[1], C.R. Young[1], J. Tingling[2], X.J. Zhou[2,4], A.R. Rimmelin[1], J.L. Leibowitz[2&3], C.J. Welsh[1,3,5]*
[1]*Veterinary Integrative Biosciences,* [2]*Microbial Pathogenesis and Immunology,*
[3]*Texas A&M Institute for Neuroscience, Texas A&M University*
[4]*College Station High School,* [5]*Women's Health in Neuroscience Program, Texas A&M Health Science Center, College Station, Texas*

Department of Veterinary Integrative Biosciences
College of Veterinary Medicine and Biomedical Sciences
Texas A&M University
Mailstop 4458
College Station, TX 77843

Supplementary Figure S1: Acute inflammation was more pronounced at the lumbar segment of spinal cord following DA-D$_S$-infection. H&E stained lumbar segments from the (a and c) control group, (b) DA-C$_L$-infected group, and (d) DA-D$_S$-infected group. (a and c) No lesions were found in the control group. (b and d) The most affected lumbar segments showing meningitis, PVC, and inflammatory foci in the gray and white matter, and representing scores 3. (e) As described in Materials and Methods, the degree of inflammation was determined at the cervical, thoracic, lumbar and sacral segments of spinal cord and compared between DA-D$_S$- and DA-C$_L$-infected groups (* $p<0.05$ by Kolmogorov-Smirnov test). Graph shows mean ± SEM, N=5 in DA-C$_L$ group and N=6 in DA-D$_S$ group. The ▼ indicates possible neuronophagia, and the ← indicates inflammation.

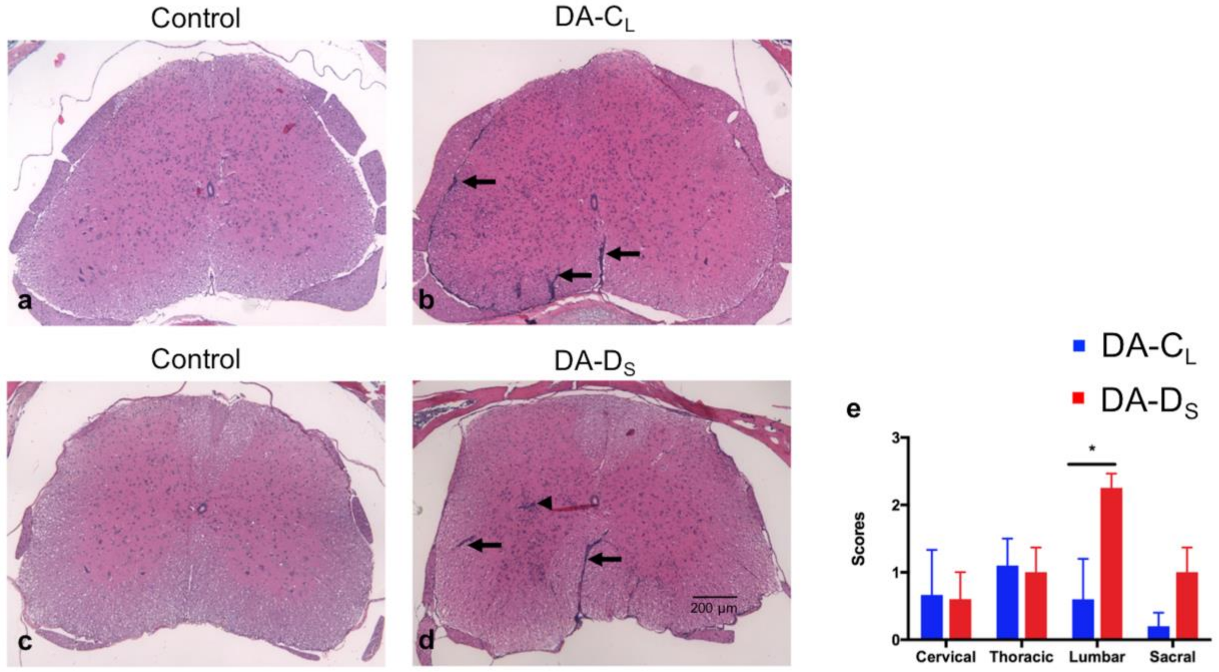

Supplementary Table S1: cDNA Synthesis Primers

Primer Sense and Position*	Use	Primer Sequence*
(-) 1170-1151	cDNA synthesis	TGG GAA CCA TTC ACC GTC TG
(-) 2538-2520	cDNA synthesis	TGA GGT CGT CGG GAC AGA A
(-) 3083-3065	cDNA synthesis	GGT AGT TTC ACG GGT TCT G
(-) 4612-4593	cDNA synthesis	GGC AAA CTG GGG GAG GAG TA
(-) 6312-6293	cDNA synthesis	CTT ACT ACG ACA GTG GCC CT
(-) 7835-7813	cDNA synthesis	AGT TTC TCT TTA AGT GTT CCT GG
3' RACE-OligoT	cDNA synthesis	GAC TCG AGT CGA CAT CGA $(T)_{17}$

*Primer positions and sequences are from Genbank Accession M20301

Supplementary Table S2: PCR products amplified for sequencing

PCR Primer	Primer Sequence	cDNA Used for PCR	Amplified cDNAs
(+)123-142	ACT CCC GAC TCC GCA CCC TA	DA (-) 1170-1151	123-1170
(-)1170-1151	TGG GAA CCA TTC ACC GTC TG		
(+)750-769	ACA CAA AGG CAG CGG AAC CC	DA (-) 2538-2520	750-1760
(-)1760-1741	GGG AGA GGA ATG CGG ATG TG		
(+)1087-1106	CCA GAT GTG TGC CCT ATT TG	DA (-) 3083-3065	1087-3083
(-)3083-3065	GGT AGT TTC ACG GGT TCT G		
(+)1862-1881	CTC AAT TTC ACG CCG GCT CT	DA (-) 2538-2520	1862-2538
(-)2538-2520	TGA GGT CGT CGG GAC AGA A		
(+)2806-2825	TCC CCT ACT CAC TAT CGC CA	DA (-) 4612-4593	2806-4612
(-)4612-4593	GGC AAA CTG GGG GAG GAG TA		
(+)2946-2965	GTC TCC GCG GGA GAT GAT TT	DA (-) 4612-4593	2946-4612
(-)4612-4593	GGC AAA CTG GGG GAG GAG TA		
(+)3753-3772	CTT CTT TCC CTG GCC TGT GT	DA (-) 6312-6293	3753-5839
(-)5856-5839	CTC AAC TCT CAC GGG CGA A		
(+)4229-4248	CCC TGT GCA GTC GGT TTT TC	DA (-) 6312-6293	4229-6312
(-)6312-6293	CTT ACT ACG ACA GTG GCC CT		
(+)6031-6050	CCT GAC AAG GCT GAA GTG AC	DA (-) 7835-7813	6031-7835
(-)7835-7813	AGT TTC TCT TTA AGT GTT CCT GG		
(+)6031-6050	CCT GAC AAG GCT GAA GTG AC	3' RACE	6031-poly(A)
(-)3'RACE	GAC TCG AGT CGA CAT CGA		
(+)6066-6079	GCT CCG TGC CCA CC	3'RACE 6031-poly(A)	6066-poly(A)
(-)3'RACE	GAC TCG AGT CGA CAT CGA		

Supplementary Table S3: Sequencing Primer

Primer Name Position	Polarity	Sequence (5’->3’)	Length
DA (+)123-142	+sense	ACT CCC GAC TCC GCA CCC TA	20
DA (-)450-431	-sense	TCA CAT AAT CGG GGA GAC AT	20
DA (-)625-606	-sense	AAG GAA GGG GCA ACA CAT AC	20
DA (+)750-769	+sense	ACA CAA AGG CAG CGG AAC CC	20
DA (+)1087-1106	+sense	CCA GAT GTG TGC CCT ATT TG	20
DA (-)1170-1151	-sense	TGG GAA CCA TTC ACC GTC TG	20
DA (+)1428-1447	+sense	CCC CCA AAA CAA CGG ACA AT	20
DA (+)1737-1756	+sense	CTC CCA CAT CCG CAT TCC TC	20
DA (-)1760-1741	-sense	GGG AGA GGA ATG CGG ATG TG	20
DA (+) 1862-1881	+sense	CTC AAT TTC ACG CCG GCT CT	20
DA (-) 2538-2520	-sense	TGA GGT CGT CGG GAC AGA A	19
DA (+)2806-2825	+sense	TCC CCT ACT CAC TAT CGC CA	20
DA (+)2946-2965	+sense	GTC TCC GCG GGA GAT GAT TT	20
DA (-)3083-3065	-sense	GGT AGT TTC ACG GGT TCT G	19
DA (-)3538-3520	-sense	CAG CAC CGA CGA GCC ACA T	19
DA (+)3753-3772	+sense	CTT CTT TCC CTG GCC TGT GT	20
DA (-)3905-3886	-sense	CCG TGA ACC TTG TAG TCA AA	20
DA (+)4229-4248	+sense	CCC TGT GCA GTC GGT TTT TC	20
DA (-)4612-4593	-sense	GGC AAA CTG GGG GAG GAG TA	20
DA (+)4740-4759	+sense	ACG TGA GGC CAA TGA AGG TT	20
DA (-)5328-5309	-sense	AAC AGC AGG GTA ATG GGC AA	20
DA (-)5856-5837	-sense	CTC AAC TCT CAC GGG CGA A	19
DA (+)6031-6050	+sense	CCT GAC AAG GCT GAA GTG AC	20
DA (-)6312-6293	-sense	CTT ACT ACG ACA GTG GCC CT	20
DA (+)6660-6679	+sense	TCA TGA TGT TTT CCA ACC CA	20
DA (+)7766-7787	+sense	CTC AAA TGG ATG CTG TCA ACT T	22
DA (-)7835-7813	-sense	AGT TTC TCT TTA AGT GTT CCT GG	23
